# A database of high-pressure crystal structures from hydrogen to lanthanum

**DOI:** 10.1038/s41597-024-03447-1

**Published:** 2024-07-12

**Authors:** Federico Giannessi, Simone Di Cataldo, Santanu Saha, Lilia Boeri

**Affiliations:** 1 Dipartimento di Scienze Fisiche e Chimiche, Università degli Studi dell’Aquila, Via Vetoio 40, 67100 L’Aquila, Italy; 2 Enrico Fermi Research Center, Via Panisperna 89 A, 00184 Rome, Italy; 3 Dipartimento di Fisica, Sapienza Università di Roma, 00185 Rome, Italy; 4 Institut für Festkörperphysik, Wien University of Technology, 1040 Wien, Austria; 5 Institute of Theoretical and Computational Physics, Graz University of Technology, NAWI Graz, 8010 Graz, Austria; 6 Department of Physics, University of Oxford, Parks Rd, Oxford, OX1 3PU UK; 7 Institut de Recherche sur les Céramiques (IRCER), UMR CNRS 7315-Université de Limoges, Limoges, 87068 France

**Keywords:** Structure of solids and liquids, Mineralogy

## Abstract

This paper introduces the *HEX (High-pressure Elemental Xstals)* database, a complete database of the ground-state crystal structures of the first 57 elements of the periodic table, from H to La, at 0, 100, 200 and 300 GPa. HEX aims to provide a unified reference for high-pressure research, by compiling all available experimental information on elements at high pressure, and complementing it with the results of accurate evolutionary crystal structure prediction runs based on Density Functional Theory. Besides offering a much-needed reference, our work also serves as a benchmark of the accuracy of current *ab-initio* methods for crystal structure prediction. We find that, in 98% of the cases in which experimental information is available, *ab-initio* crystal structure prediction yields structures which either coincide or are degenerate in enthalpy to within 300 K with experimental ones. The main manuscript contains synthetic tables and figures, while the Crystallographic Information File (cif) for all structures can be downloaded from the related figshare online repository.

## Background & Summary

The advent of 21st century marks a pivotal moment for high-pressure research: advancements diamond anvil cells design and *in-situ* characterization techniques^1–4^ gave access to the realm of multi-megabar pressures, revealing unexpected and fascinating phenomena, such as high-temperature conventional superconductivity in H_3_S^5,6^, LaH_10_^7,8^ and other superhydrides^2^, metal-insulator transition in elemental sodium^9^, self-ionization of boron^10^, electride behavior in alkali metals^11^, noble-gas solids^12^, etc.

Until the turn of the century, knowledge on the behaviour of matter at high pressure was limited and based on indirect evidence. The general expectation was that all matter would tend to become homogeneous and metallic to maximize the electronic kinetic energy. However, experiments over the last 30 years revealed a much more varied behaviour defying this naïve expectation. Compounds at high pressures often adopt exotic crystal structures, whose stoichiometries, motifs and moieties defy fundamental chemical concepts, such as valence and electronegativity, which govern the behaviour of matter at ambient pressure^13^. The most striking examples of this so-called *forbidden chemistry* are highlighted in several excellent review papers^14–18^, which also offer a glimpse on the underlying physical mechanisms, such as polymerization, rearrangements of atomic orbital energies, interstitial charge localization, etc.

*Ab-initio* calculations based on Density Functional Theory (DFT) have played a pivotal role in high-pressure research. Nowadays, these methods permit not only to describe known phases from a microscopic quantum-mechanical viewpoint, but also to predict new structures and properties. The famous Maddox paradox, according to which the quantum mechanical methods for material modelling cannot be considered fully predictive, unless they can predict crystal structures from the knowledge of the sole chemical composition, has finally been overcome^19^. In fact, modern techniques for crystal structure prediction have proven their predictive power over a variety of systems, with an astounding agreement with experimental observations^20^. These techniques utilize clever optimization strategies to identify the global and local minima of the potential energy surface (PES) associated with a given set of atoms, which correspond to the ground-state and metastable structures^21^, respectively. Commonly-employed methods include simulated annealing, *ab-initio* random structure search^22^, metadynamics^23^, minima hopping^24,25^, evolutionary algorithms^26–28^, particle swarm optimization^29,30^, etc.

Indeed, thanks to the increasing integration between experimental and computational methodologies, the knowledge on high-pressure crystal structures has experienced significant advancements in recent years. However, the relative information is still largely incomplete, and spread over several databases and publications, whose standards vary significantly. A large portion of these sources is either unaccessible due to paywalls or rely on outdated conventions. Even for the most basic systems, such as mono-elemental solids, it is frequently challenging to find complete crystal structure information for the entire range of experimentally-accessible pressures, which nowadays exceed 400 GPa. In fact, particularly at higher pressures, the only available crystal structures information derives from computational predictions, which significantly differ in terms of breadth and accuracy.

The aim of the *High-pressure Elemental Xstals* database^31^ (*HEX* database) is to provide a single open-access, easily accessible and well-organized database containing the crystal structures of the first 57 elements of the periodic table (Hydrogen-Lanthanum) at pressures of 0, 100, 200 and 300 GPa. The database has been constructed compiling all available literature, and comparing with the results of highly-accurate evolutionary crystal structure prediction calculations^26^, based on plane-wave pseudopotential Density Functional Theory (DFT) total energies. Our choice to exclude elements beyond lanthanum is motivated by the need to maintain a consistent accuracy throughout the database: Elements in the lanthanide and actinide series have been excluded, due to the inadequacy of the pseudopotential approximation for elements with open *f*-shells, while other heavy elements were discarded, because significant spin-orbit interaction may introduce further sources of inaccuracy in the calculations. In order to maintain the computational cost manageable, our evolutionary crystal structure prediction runs employ 8-atoms unit cells, and neglect zero-point energy (ZPE) corrections, which should however be negligible for elements beyond the first rows.

The primary aim of this work is to provide a complete and accurate reference for researchers in various fields. Moreover, by presenting a systematic comparison of high-quality crystal structure prediction results with literature data, the HEX database^31^ also gives an extensive benchmark of the accuracy of crystal structure prediction methods on elemental crystal structures, which nicely complements existing blind tests on molecules^32^. We find that evolutionary algorithm (EA) predictions reproduce known experimental results in over 95% of the cases; most of the observed deviations can be attributed to the use of too small unit cells.

## Methods

Data contained in the *HEX* database^31^ were generated by combining literature data with results of evolutionary crystal structure prediction runs.

We performed a thorough screening of the available literature to identify the ground-state crystal structures of the first 57 elements of the periodic table (H-La), at 0, 100, 200 and 300 GPa. Moreover, we performed unconstrained *ab-initio* EA searches for each element and pressure, as explained below. The structures obtained from the two sources underwent a final relaxation and symmetrization employing the same convergence criteria. This allowed us to compare the total energies/enthalpies to determine a single ground-state crystal structure for each element and pressure; taken all together, these structures form the first sub-database – (Database Ground-State). We also created two other sub-databases, one containing all structures predicted by EA runs – (Database Evolutionary Algorithm), and the other containing all literature (LIT) structures which turned out to be less energetically favorable than the EA ones (Database Mismatch). The content and structure of the three sub-databases is described in detail in the Data Records section; here we describe in detail the generation procedure.

- **EA-generated structures:** The bulk of our work involved crystal structure prediction runs for the first 57 elements of the periodic table (H-La), over a wide range of pressures. We employed evolutionary algorithms as implemented in the *Universal Structure Predictor: Evolutionary Xtallography* (USPEX) code^33–35^. Structural searches for each element were carried out at 0, 100, 200, 300 GPa to identify the lowest-enthalpy structure. The underlying structural relaxations and total energy calculations are based on Density Functional Theory (DFT), as implemented in the *Vienna Ab Initio Simulation Package* (VASP)^36,37^. We employed Projector Augmented Wave pseudopotentials^38,39^ part of the standard VASP distribution, and Perdew-Burke-Ernzerhof exchange-correlation functional^40^. For reciprocal **k**-space integration we used uniform Monkhorst-Pack grids^41^ with Methfessel-Paxton smearing^42^ (See Table 1 for further details).

**Table 1:** Computational details of the multi-step DFT relaxation procedure employed in evolutionary crystal structure prediction runs; ENMIN/ENMAX indicate the minimum/maximum kinetic energy cutoff values reported in the VASP pseudopotential files; Δ*E* and Δ*F* indicate the total energy and force convergence criteria, respectively.

Step	Plane wave Cutoff (eV)	Smearing (eV)	k-point Spacing (2*π* × Å^−1^)	Δ*E* (eV)	Δ*F* (eV/Å)
1	ENMIN	0.10	0.13	10^−2^	10^−1^
2	ENMAX	0.08	0.11	10^−3^	10^−2^
3	500	0.07	0.09	5×10^−4^	5×10^−3^
4	600	0.07	0.07	10^−4^	10^−3^
5	600	0.06	0.04	10^−5^	10^−3^
final	600	0.06	0.04	10^−5^	10^−3^

The final row of the table contains the settings used for the final relaxation of the EA-generated structures as well as literature structures.
  For each combination of element and pressure, we performed EA searches with an 8-atom unit cell. The first generations contained 40 structures (Individuals), while each of the following generation contained 20 structures. Each individual was fully relaxed, following a five-step relaxation procedure with increasing accuracy; the relevant parameters are summarized in Table 1. Crystal structure prediction runs lasted for a maximum of 20 generations, and were considered converged when the the lowest-enthalpy structure remained the same for 7 consecutive generations. Once the evolutionary algorithm search was converged, we collected the ten lowest-enthalpy structures for each element and pressure. These structures underwent a final relaxation, with tighter criteria listed in the *final* row of Table 1, and finally symmetrized, using the FINDSYM algorithm by Stokes *et al*.^43,44^, with a tolerance criterion of 0.2 Å. The lowest-enthalpy structure after symmetrization for each element and pressure was selected as the EA ground-state structure. If, after the *final* relaxation and symmetrization, we found more than one structure to be degenerate in enthalpy within 26 meV (i.e. *k*_*B*_*T* for *T* = 300 *K*), we selected the highest-symmetry one.
- **Literature search:** We performed a thorough screening of the existing literature on the crystal structures of the first 57 elements of the periodic table (H-La), at 0, 100, 200 and 300 GPa. We chose experimental references rather than theoretical ones, when available, and more recent papers were selected in favour of older ones. Our bibliographic search was performed as comprehensively as possible using multiple queries and strategies. However, we cannot rule out that we may have missed some references.

The experimental structures at ambient pressure were extracted from the American Mineralogist Crystal Structure Database^45^, while information on higher pressure was obtained from multiple sources.

All references, along with the indication on whether they refer to a theoretical or experimental work, are reported in the *Ref* column of Tables 2–10. Once identified, structures extracted from literature underwent a single run of structural relaxation, with the same settings used for the *final* relaxation of EA-generated structures before their energies were compared with the EA results. The parameters reported in the tables refer to this *final* relaxation.

**Table 2:** Ground-state database (DB_GS) at 0 GPa.

Z	Element	Space group	Volume (Å^3^/*atom*)	Wyckoff positions	Source	Ref.
1	H	194	14.77	*(4 f) z = −*0*.17853*	lit.	exp.^53^
2	He	194	17.30	*(2c)*	*	exp.^54^
3	Li	229	21.27	*(2a)*	lit.	exp.^55^
4	Be	194	7.91	*(2c)*	*	exp.^56^
5	B	166	7.26	*(18 h) x = −0.21455 z = 0.22487*	lit.	exp.^57^
				*(18 h) x = 0.19695 z = 0.02424*		
6	C	191	10.34	*(2d)*	*	exp.^58^
7	N	194	33.54	*(4 f) z = −0.33949*	lit.	exp.^59^
8	O	12	14.58	*(4i) x = −0.14455 z = 0.12053*	lit.	exp.^60^
				*(4i) x = 0.20159 z = 0.186*26		
				*(8j) x = *0*.02857 y = 0.23724 z = 0.15337*		
9	F	12	17.90	*(4i) x = 0.05400 z = 0.10073*	lit.	exp.^61^
				*(4i) x* = *−0.49221 z* = *−0.39902*		
10	Ne	225	22.36	*(4a)*	*	exp.^58^
11	Na	229	37.71	*(2a)*	lit.	exp.^58^
12	Mg	194	23.09	*(2d)*	*	exp.^58^
13	Al	225	16.53	*(4a)*	*	exp.^58^
14	Si	227	20.46	*(8b)*	*	exp.^58^
15	P	164	23.84	*(2d) z = 0.12144*	*	exp.^62^
16	S	70	34.54	*(32 h) x = 0.20642 y = 0.05407 z = 0.10800*	lit.	exp.^63^
				*(32 h) x = −0.24429 y = 0.02723 z = 0.47620*		
				*(32 h) x = −0.17438 y = 0.48401 z = 0.03896*		
				*(32 h) x = 0.37513 y = 0.09684 z = 0.04511*		
17	Cl	64	34.21	*(8 f) y = −0.11492 z = −0.10573*	lit.	exp.^64^
18	Ar	225	45.84	*(4a)*	*	exp.^58^
19	K	229	73.39	*(2a)*	lit.	exp.^58^
20	Ca	225	42.58	*(4a)*	*	exp.^58^
21	Sc	194	24.52	*(2d)*	*	exp.^58^
22	Ti	194	17.42	*(2c)*	*	exp.^58^
23	V	229	13.50	*(2a)*	*	exp.^58^
24	Cr	229	11.46	*(2a)*	*	exp.^58^
25	Mn	217	10.74	*(2a)*	lit.	exp.^65^
				*(8c) x = 0.18194*		
				*(24 g) x = 0.14325 z = 0.46199*		
				*(24 g) x = 0.41117 z = 0.21894*		
26	Fe*	229	11.77	*(2a)*	*	exp.^66^
27	Co*	194	10.20	*(2c)*	*	exp.^58^
28	Ni*	225	10.76	*(4a)*	*	exp.^58^
29	Cu	225	12.00	*(4a)*	*	exp.^58^
30	Zn	194	15.25	*(2d)*	*	exp.^58^
31	Ga	64	20.53	*(8 f) y = 0.34222 z = 0.41786*	*	exp.^67^
32	Ge	227	24.15	*(8a)*	*	exp.^68^
33	As	166	22.93	*(6c)*	*	exp.^69^
34	Se	14	39.00	*(4e) x = 0.06913 y = 0.49655 z = 0.24516*	lit.	exp.^70^
				*(4e) x = 0.05854 y = 0.66250 z = 0.35690*		
				*(4e) x = −0.21494 y = −0.36324 z = −0.46970*		
				*(4e) x = −0.40449 y = −0.20629 z = −0.45721*		
				*(4e) x = 0.40877 y = −0.30798 z = −0.48887*		
				*(4e) x = −0.47828 y = −0.27649 z = 0.32991*		
				*(4e) x = −0.31228 y = −0.45720 z = 0.23086*		
				*(4e) x = −0.01374 y = −0.40631 z = 0.14476*		
35	Br^†^	64	30.88	*(8 f) y = 0.15416 z = 0.11932*	lit.	exp.^64^
36	Kr	225	66.44	*(4a)*	*	exp.^58^
37	Rb	229	91.09	*(2a)*	lit.	exp.^58^
38	Sr	225	54.97	*(4a)*	*	exp.^58^
39	Y	194	32.53	*(2c)*	*	exp.^58^
40	Zr	194	23.44	*(2d)*	*	exp.^58^
41	Nb	229	18.16	*(2a)*	*	exp.^58^
42	Mo	229	15.83	*(2a)*	*	exp.^58^
43	Tc	194	14.21	*(2c)*	lit.	exp.^58^
44	Ru	194	13.71	*(2c)*	*	exp.^58^
45	Rh	225	14.08	*(4a)*	*	exp.^58^
46	Pd	225	15.30	*(4a)*	*	exp.^58^
47	Ag	225	18.00	*(4a)*	*	exp.^71^
48	Cd	194	22.79	*(2c)*	lit.	exp.^58^
49	In	139	27.43	*(2a)*	lit.	exp.^72^
50	Sn	227	36.87	*(8a)*	*	exp.^72^
51	Sb	166	32.13	*(6c) z = 0*.26*654*	*	exp.^69^
52	Te	152	34.99	*(3a) x = *0*.73640*	lit.	exp.^73^
53	I^†^	64	41.31	*(8 f) y = −0.16897 z = 0.37788*	lit.	exp.^58^
54	Xe	225	88.43	*(4a)*	*	exp.^58^
55	Cs	229	117.16	*(2a)*	lit.	exp.^58^
56	Ba	229	64.07	*(2a)*	*	exp.^58^
57	La	194	37.56	*(2a)*	lit.	exp.^58^
				*(2d)*		

The symbols in the *Source* column indicate: (i) * the structure is *matching*; (ii) *-* no reference could be found in literature; (iii) *lit*./*ea*. the ground-state structure originates from literature/evolutionary algorithm. In the *Ref* column, (i) *th*. (*exp*.) specifies whether the literature source is computational (experimental), or (ii) *-* missing; (iii) Fe*, Co* and Ni* indicate that the calculation is spin-polarized with a magnetic moment of 2.22, 1.74, 0.606 *a.u*. respectively^74^ and (iv) *Br*^†^ and *I*^†^, indicate that the calculation employed the opt88-vdW *xc* functional^75^.

**Table 3:** Ground-state database (DB_GS) at 100 GPa.

Z	Element	Space group	Volume (Å^3^/*atom*)	Wyckoff positions	Source	Ref.
1	H	176	2.31	*(4 f) z = −0.37200*	lit.	th.^76^
				*(6 h) x = 0.09840 y = 0.39006*		
				*(6 h) x = 0.19824 y = 0*.26*598*		
2	He	194	3.46	*(2c)*	—	—
3	Li	64	6.00	*(8 f) y = −*0*.174*26 *z = 0.43844*	lit.	th.^51^
				*(16 g) x = 0.33351 y = 0.10864 z = 0.34339*		
4	Be	194	5.27	*(2c)*	*	th.^77^
5	B	64	5.00	*(8 f) y = −0.15747 z = −0.08761*	*	exp.^78^
6	C	227	4.83	*(8b)*	*	th.^79^
7	N	199	5.52	*(8a) x = 0.32248*	*	th.^50^
8	O	15	6.00	*(8 f) x = 0.09795 y = 0.29000 z = 0.21503*	*	th.^80^
9	F	64	6.29	*(8 f) y = 0.34595 z = 0.11698*	*	th.^81^
10	Ne	225	6.71	*(4a)*	*	exp.^82^
11	Na	225	10.78	*(4a)*	*	exp.^83^
12	Mg	229	11.25	*(2a)*	*	th.^84^
13	Al	225	10.34	*(4a)*	*	exp.^85^
14	Si	225	9.19	*(4a)*	*	exp.^86^
15	P	221	9.95	*(1a)*	*	exp.^87^
16	S	166	9.99	*(3b)*	ea.	exp.^46^
17	Cl	64	11.40	*(8 f) y = 0.31818 z = −0.37903*	*	th.^81^
18	Ar	194	12.62	*(2d)*	—	—
19	K	64	11.81	*(8d) x = −0.28496*	*	th.^88^
				*(8 f) y = 0.32494 z = 0.32775*		
20	Ca	96	12.44	*(8b) x = −0.01497 y = 0.32129 z = −0.09575*	lit.	th.^89^
21	Sc	88	12.11	*(16 f) z = −0.27743 y = −0.19917 z = 0.47208*	*	exp.^90^
22	Ti	229	10.77	*(2a)*	lit.	th.^91^
23	V	229	9.85	*(2a)*	*	th.^92^
24	Cr	229	9.06	*(2a)*	—	—
25	Mn	194	8.48	*(2c)*	*	exp.^93^
26	Fe	194	8.18	*(2c)*	*	exp.^94^
27	Co	194	8.15	*(2c)*	lit.	exp.^95^
28	Ni	225	8.31	*(4a)*	*	th.^48^
29	Cu	225	8.67	*(4a)*	—	—
30	Zn	194	9.64	*(2c)*	*	exp.^96^
31	Ga	225	10.93	*(4a)*	*	th.^47^
32	Ge	191	11.88	*(1a)*	lit.	exp.^96^
33	As	229	12.02	*(2a)*	*	th.^97^
34	Se	166	12.72	*(3b)*	*	exp.^98^
35	Br	139	23.08	*(2a)*	lit.	th.^99^
36	Kr	194	15.58	*(2c)*	—	—
37	Rb	64	15.00	*(8d) x = 0.21706*	*	th.^88^
				*(8 f) y = −0.32320 z = −0.17520*		
38	Sr	62	14.96	*(4c) x = 0.17310 z = −0.07136*	—	—
39	Y	70	14.66	*(16 g) z = 0.43748*	lit.	exp.^100^
40	Zr	229	13.82	*(2a)*	*	exp.^101^
41	Nb	229	13.00	*(2a)*	*	th.^102^
42	Mo	229	12.48	*(2a)*	*	th.^102^
43	Tc	194	11.73	*(2d)*	*	th.^103^
44	Ru	194	11.26	*(2c)*	*	th.^104^
45	Rh	225	11.26	*(4a)*	—	—
46	Pd	225	11.61	*(4a)*	*	th.^105^
47	Ag	225	12.28	*(4a)*	—	—
48	Cd	194	13.52	*(2d)*	—	—
49	In	225	15.07	*(4a)*	ea.	th.^47^
50	Sn	139	15.89	*(2b)*	*	th.^106^
51	Sb	229	16.39	*(2a)*	—	—
52	Te	225	16.53	*(4a)*	*	th.^107^
53	I	225	17.35	*(4a)*	—	—
54	Xe	194	20.05	*(2c)*	—	—
55	Cs	194	19.00	*(2a)*	*	th.^108^
				*(2c)*		
56	Ba	194	18.73	*(2d)*	—	—
57	La	225	16.73	*(4a)*	*	exp.^109^

NThe symbols in the *Source* column indicate: (i) * the structure is *matching*; (ii) *-* no reference could be found in literature; (iii) *lit*./*ea*. the ground-state structure originates from literature/evolutionary algorithm. In the *Ref* column, (i) *th*. (*exp*.) specifies whether the literature source is computational (experimental), or (ii) *-* missing.

**Table 4:** Ground-state database (DB_GS) at 200 GPa.

Z	Element	Space group	Volume (Å^3^/*atom*)	Wyckoff positions	Source	Ref.
1	H	15	1.73	*(8 f) x = 0.26735 y = 0.42295 z = 0.24415*	lit.	th.^76^
				*(8 f) x = 0.15706 y = 0.30406 z = 0.22058*		
				*(4e) y = −0.35218*		
				*(4e) y = −0.10217*		
2	He	194	2.70	*(2c)*	—	—
3	Li	64	4.48	*(8 f) y = −0.44022 z = −0.33050*	lit.	th.^51^
				*(16 g) x = 0.14346 y = 0.16867 z = 0.06475*		
				*(16 g) x = −0.28580 y = 0.39461 z = 0.34794*		
				*(16 g) x = −0.07142 y = 0.13770 z = −0.37225*		
4	Be	194	4.33	*(2d)*	*	th.^77^
5	B	64	4.34	*(8 f) y = 0.34220 z = 0.41304*	*	exp.^78^
6	C	227	4.34	*(8b)*	*	th.^79^
7	N	113	4.64	*(4e) x = −0.33587 z = 0.32228*	ea.	th.^50^
				*(4d) z = −0.16015*		
8	O	12	5.08	*(4i) x = 0.22601 z = −0.20344*	*	th.^80^
				*(4i) x = −0.29538 z = −0.20316*		
				*(8j) x = −0.46534 y = 0.25994 z = 0.20327*		
9	F	64	5.28	*(8 f) y = −0.16484 z = −0.11903*	*	th.^81^
10	Ne	225	5.54	*(4a)*	*	exp.^82^
11	Na	62	8.00	*(4c) x = 0.32391 z = −0.08020*	ea.	exp.^9^
				*(4c) x = 0.48576 z = 0.30971*		
12	Mg	229	9.02	*(2a)*	*	th.^84^
13	Al	225	8.62	*(4a)*	lit.	exp.^85^
14	Si	225	7.81	*(4a)*	*	exp.^86^
15	P	191	7.90	*(1b)*	*	exp.^110^
16	S	166	8.13	*(3b)*	*	exp.^111^
17	Cl	71	9.07	*(2a)*	*	th.^81^
18	Ar	194	10.20	*(2c)*	—	—
19	K	64	9.73	*(8d) x = −0.21662*	*	th.^88^
				*(8 f) y = 0.32384 z = 0.17522*		
20	Ca	62	9.30	*(4c) x = 0.33247 z = −0.39914*	*	exp.^112^
21	Sc	14	9.22	*(4e) x = −0.24789 y = −0.08441 z = 0.29798*	ea.	exp.^90^
22	Ti	229	8.72	*(2a)*	*	th.^91^
23	V	166	8.39	*(3a)*	*	th.^92^
24	Cr	229	7.97	*(2a)*	—	—
25	Mn	194	7.57	*(2d)*	*	exp.^93^
26	Fe	194	7.29	*(2c)*	*	exp.^94^
27	Co	225	7.24	*(4a)*	*	exp.^95^
28	Ni	225	7.34	*(4a)*	ea.	th.^48^
29	Cu	225	7.57	*(4a)*	—	—
30	Zn	194	8.28	*(2c)*	*	exp.^96^
31	Ga	225	9.21	*(4a)*	*	th.^47^
32	Ge	194	9.80	*(2c)*	*	exp.^96^
33	As	229	10.15	*(2a)*	*	th.^97^
34	Se	229	10.62	*(2a)*	*	exp.^96^
35	Br	225	11.20	*(4a)*	*	th.^99^
36	Kr	194	12.63	*(2d)*	—	—
37	Rb	194	12.18	*(2a)*	*	th.^88^
				*(2d)*		
38	Sr	194	11.92	*(2d)*	—	—
39	Y	70	11.64	*(16e) x = 0.43748*	*	th.^113^
40	Zr	229	11.32	*(2a)*	—	—
41	Nb	229	11.17	*(2a)*	*	th.^102^
42	Mo	229	10.97	*(2a)*	*	th.^102^
43	Tc	194	10.35	*(2c)*	—	—
44	Ru	194	10.09	*(2c)*	*	th.^104^
45	Rh	225	10.06	*(4a)*	—	—
46	Pd	225	10.25	*(4a)*	—	—
47	Ag	225	10.69	*(4a)*	—	—
48	Cd	194	11.50	*(2c)*	—	—
49	In	139	12.68	*(2b)*	*	th.^47^
50	Sn	194	13.40	*(2c)*	*	th.^106^
51	Sb	229	13.80	*(2a)*	—	—
52	Te	225	13.96	*(4a)*	*	th.^107^
53	I	225	14.37	*(4a)*	—	—
54	Xe	194	16.24	*(2c)*	—	—
55	Cs	225	15.60	*(4a)*	*	th.^108^
56	Ba	194	15.67	*(2d)*	—	—
57	La	225	14.14	*(4a)*	*	exp.^109^

The symbols in the *Source* column indicate: (i) * the structure is *matching*; (ii) *-* no reference could be found in literature; (iii) *lit*./*ea*. the ground-state structure originates from literature/evolutionary algorithm. In the *Ref* column, (i) *th*. (*exp*.) specifies whether the literature source is computational (experimental), or (ii) *-* missing.

**Table 5:** Ground-state database (DB_GS) at 300 GPa.

Z	Element	Space group	Volume (Å^3^/*atom*)	Wyckoff positions	Source	Ref.
1	H	64	1.45	*(8 f) x = 0.00366 z = 0.13499*	lit.	th.^76^
				*(4 f) x = 0.13176 z = 0.45360*		
				*(4 f) x = 0.26798 z = 0.31709*		
2	He	194	2.32	*(2d)*	—	—
3	Li	64	3.80	*(8 f) y = 0.05146 z = 0.17196*	lit.	th.^51^
				*(16 g) x = −0.35620 y = 0.17042 z = −0.44172*		
				*(16 g) x = 0.21371 y = 0.39288 z = −0.15060*		
				*(16 g) x = 0.42870 y = 0.13685 z = 0.12675*		
4	Be	194	3.81	*(2c)*	*	th.^114^
5	B	64	3.93	*(8 f) y = 0.157750 z = −0.08527*	—	—
6	C	194	4.00	*(4e) z = −0.09278*	—	—
				*(4 f) z = −0.34395*		
7	N	64	4.18	*(8 f) y = 0.10538 z = −0.10004*	ea.	th.^50^
8	O	166	4.55	*(6c) z = −0.06915*	—	—
9	F	64	4.72	*(8 f) y = −0.32735 z = −0.37974*	*	th.^81^
10	Ne	225	4.91	*(4a)*	—	—
11	Na	194	6.68	*(2a)*	*	exp.^9^
				*(2d)*		
12	Mg	229	7.84	*(2a)*	*	th.^84^
13	Al	194	7.57	*(2c)*	*	exp.^115^
14	Si	225	7.00	*(4a)*	—	—
15	P	220	6.84	*(16c) x = 0.47267*	—	—
16	S	166	7.15	*(3a)*	*	th.^116^
17	Cl	139	7.90	*(2a)*	*	th.^81^
18	Ar	194	8.92	*(2c)*	—	—
19	K	194	8.47	*(2a)*	*	th.^88^
				*(2d)*		
20	Ca	62	8.07	*(4c)*	—	—
21	Sc	180	7.83	*(3c)*	lit.	exp.^90^
22	Ti	229	7.61	*(2a)*	*	exp.^117^
23	V	229	7.57	*(2a)*	*	th.^92^
24	Cr	229	7.28	*(2a)*	—	—
25	Mn	194	6.91	*(2d)*	—	—
26	Fe	194	6.73	*(2c)*	*	exp.^94^
27	Co	225	6.67	*(4a)*	—	—
28	Ni	225	6.73	*(4a)*	—	—
29	Cu	225	6.91	*(4a)*	—	—
30	Zn	194	7.49	*(2d)*	—	—
31	Ga	225	8.27	*(4a)*	—	—
32	Ge	194	8.74	*(2c)*	*	exp.^96^
33	As	229	9.07	*(2a)*	—	—
34	Se	139	9.44	*(2a)*	*	exp.^96^
35	Br	225	9.91	*(4a)*	*	th.^118^
36	Kr	194	11.09	*(2d)*	—	—
37	Rb	194	10.83	*(2a)*	lit.	th.^88^
				*(2d)*		
38	Sr	194	10.61	*(2c)*	—	—
39	Y	70	10.25	*(16e) x = −0.18749*	lit.	th.^52^
40	Zr	229	10.05	*(2a)*	—	—
41	Nb	229	10.04	*(2a)*	*	th.^102^
42	Mo	229	9.99	*(2a)*	*	th.^102^
43	Tc	194	9.52	*(2c)*	*	th.^103^
44	Ru	194	9.32	*(2d)*	*	th.^104^
45	Rh	225	9.30	*(4a)*	—	—
46	Pd	225	9.43	*(4a)*	—	—
47	Ag	225	9.76	*(4a)*	—	—
48	Cd	194	10.52	*(2c)*	—	—
49	In	139	11.38	*(2a)*	*	th.^47^
50	Sn	194	12.01	*(2d)*	—	—
51	Sb	229	12.37	*(2a)*	—	—
52	Te	225	12.49	*(4a)*	—	—
53	I	139	12.73	*(2b)*	—	—
54	Xe	194	14.19	*(2c)*	—	—
55	Cs	225	13.87	*(4a)*	—	—
56	Ba	194	14.03	*(2c)*	—	—
57	La	139	12.61	*(2a)*	—	—

The symbols in the *Source* column indicate: (i) * the structure is *matching*; (ii) *-* no reference could be found in literature; (iii) *lit*./*ea*. the ground-state structure originates from literature/evolutionary algorithm. In the *Ref* column, (i) *th*. (*exp*.) specifies whether the literature source is computational (experimental), or (ii) *-* missing.

**Table 6:** Evolutionary algorithm database (DB_EA) at 0 GPa.

Z	Element	Space group	Volume (Å^3^/*atom*)	Wyckoff positions	ΔH_EA−GS_ (meV/atom)	Ref.
1	H	4	12.19	*(2a) x = 0.05164 y = 0.04451 z = −0.20950*	<26	exp.^53^
				*(2a) x = −0.44063 y = 0.45391 z = −0.30440*		
				*(2a) x = −0.04582 y = −0.05913 z = −0.28855*		
				*(2a) x = 0.46408 y = −0.45833 z = −0.20914*		
2	He	194	17.30	*(2c)*	0	exp.^54^
3	Li	139	20.20	*(2b)*	<26	exp.^55^
4	Be	194	7.91	*(2c)*	0	exp.^56^
5	**B**	**166**	**7.33**	***(6c) z = −0.06626***	**175.9**	**exp.**^57^
				***(6c) z = −0.19851***		
6	C	191	10.34	*(2d)*	0	exp.^58^
7	N	4	27.47	*(2a) x = 0.10164 y = −0.21303 z = 0.37309*	<26	exp.^59^
				*(6c) x = −0.39456 y = −0.27416 z = −0.36853*		
				*(6c) x = 0.38647 y = 0.28405 z = 0.14917*		
				*(2a) x = −0.10394 y = 0.23297 z = −0.15331*		
8	O	13	13.71	*(4 g) x = 0.14641 y = 0.17573 z = 0.28949*	<26	exp.^60^
9	F	15	18.36	*(8 f) x = −0.39931 y = 0.03411 z = 0.00111*	<26	exp.^61^
				*(8 f) x = 0.46870 y = 0.39850 z = 0.49744*		
10	Ne	225	22.36	*(4a)*	0	exp.^58^
11	Na	225	38.46	*(4a)*	<26	exp.^58^
12	Mg	194	23.09	*(2d)*	0	exp.^58^
13	Al	225	16.53	*(4a)*	0	exp.^58^
14	Si	227	20.46	*(8b)*	0	exp.^58^
15	P	164	23.84	*(2d) z = 0.12144*	0	exp.^62^
16	**S**	**82**	**37.93**	***(8 g) x = 0.34946 y = 0.21652 z = 0.19856***	**32.4**	**exp.**^63^
				***(8 g) x = 0.27671 y = 0.35174 z = 0.42027***		
17	Cl	12	42.58	*(4i) x = −0.11504 z = −0.29223*	<26	exp.^64^
18	Ar	225	45.84	*(4a)*	0	exp.^58^
19	K	194	73.37	*(2d)*	<26	exp.^58^
20	Ca	225	42.58	*(4a)*	0	exp.^58^
21	Sc	194	24.52	*(2d)*	0	exp.^58^
22	Ti	194	17.42	*(2c)*	0	exp.^58^
23	V	229	13.50	*(2a)*	0	exp.^58^
24	Cr	229	11.46	*(2a)*	0	exp.^58^
25	**Mn**	**223**	**10.69**	***(2a)***	**62.7**	**exp.**^65^
				***(6d)***		
26	Fe*	229	11.77	*(2a)*	0	exp.^66^
27	Co*	194	10.20	*(2c)*	0	exp.^58^
28	Ni*	225	10.76	*(4a)*	0	exp.^58^
29	Cu	225	12.00	*(4a)*	0	exp.^58^
30	Zn	194	15.25	*(2d)*	0	exp.^58^
31	Ga	64	20.53	*(8 f) y = 0.34222 z = 0.41786*	0	exp.^67^
32	Ge	227	24.15	*(8a)*	0	exp.^68^
33	As	166	22.93	*(6c)*	0	exp.^69^
34	Se	5	38.51	*(4c) x = 0.29680 y = 0.21693 z = −0.09413*	<26	exp.^70^
				*(4c) x = −0.34697 y = 0.45560 z = 0.24726*		
				*(4c) x = −0.10462 y = 0.47555 z = −0.24530*		
				*(4c) x = −0.18926 y = 0.46879 z = 0.39753*		
35	Br^†^	62	36.16	*(4c) x = −0.25210 z = −0.09056*	<26	exp.^64^
				*(4c) x = −0.00218 z = 0.24949*		
36	Kr	225	66.44	*(4a)*	0	exp.^58^
37	Rb	139	184.31	*(2a)*	<26	exp.^58^
38	Sr	225	54.97	*(4a)*	0	exp.^58^
39	Y	194	32.53	*(2c)*	0	exp.^58^
40	Zr	194	23.44	*(2d)*	0	exp.^58^
41	Nb	229	18.16	*(2a)*	0	exp.^58^
42	Mo	229	15.83	*(2a)*	0	exp.^58^
43	Tc	164	14.47	*(2d) z = −0.25317*	<26	exp.^58^
44	Ru	194	13.71	*(2c)*	0	exp.^58^
45	Rh	225	14.08	*(4a)*	0	exp.^58^
46	Pd	225	15.30	*(4a)*	0	exp.^58^
47	Ag	225	18.00	*(4a)*	0	exp.^71^
48	Cd	166	23.10	*(3a)*	<26	exp.^58^
49	In	229	27.74	*(2a)*	<26	exp.^72^
50	Sn	227	36.87	*(8a)*	0	exp.^72^
51	Sb	166	32.13	*(6c) z = 0.26654*	0	exp.^69^
52	**Te**	**166**	**32.56**	***(3a)***	**46.5**	**exp**^73^.
53	I^†^	63	42.09	*(4a)*	<26	exp.^58^
				*(4c) y = −0.36584*		
54	Xe	225	88.43	*(4a)*	0	exp.^58^
55	Cs	166	113.82	*(3a)*	<26	exp.^58^
56	Ba	229	64.07	*(2a)*	0	exp.^58^
57	La	225	37.85	*(4a)*	<26	exp.^58^

In the Δ*H*_EA-GS_ column, (i) *0* indicates that the EA crystal structure is lower in enthalphy than the corresponding LIT crystal structure; (ii) <*26* that the EA crystal structure is degenerate in enthalpy with the corresponding LIT crystal structure, (iii) Otherwise, it indicates the difference in enthalpy between the ground-state crystal structure and the EA-generated crystal structure, in meV/atom. In the *Ref* column, (i) *th*. (*exp*.) indicates that the literature source is computational (experimental), or (ii) *-* missing; (iii) Fe*, Co* and Ni* indicate that the calculation is spin-polarized, with a magnetic moment of 2.22, 1.74, 0.606 *a.u*. respectively^74^ and (iv) *Br*^†^ and *I*^†^, that the calculation includes vdW interactions through the opt88-vdW exchange-correlation functional^75^.

**Table 7:** Evolutionary algorithm database (DB_EA) at 100 GPa.

Z	Element	Space group	Volume (Å^3^/*atom*)	Wyckoff positions	ΔH_EA−GS_ (meV/atom)	Ref.
1	H	62	2.31	*(4c) x = −0.24849 z = −0.10890*	<26	th.^76^
				*(4c) x = −0.41675 z = 0.14571*		
2	He	194	3.46	*(2c)*	0	—
3	Li	43	6.06	*(16b) x = 0.37357 y = 0.47947 z = 0.47349*	<26	th.^51^
				*(16b) x = 0.25385 y = 0.12473 z = 0.20675*		
4	Be	194	5.27	*(2c)*	0	th.^77^
5	B	64	5.00	*(8 f) y = −0.15747 z = −0.08761*	0	exp.^78^
6	C	227	4.83	*(8b)*	0	th.^79^
7	N	199	5.52	*(8a) x = 0.32248*	0	th.^50^
8	O	15	6.00	*(8 f) x = 0.09795 y = 0.29000 z = 0.21503*	0	th.^80^
9	F	64	6.29	*(8 f) y = 0.34595 z = 0.11698*	0	th.^81^
10	Ne	225	6.71	*(4a)*	0	exp.^82^
11	Na	225	10.78	*(4a)*	0	exp.^83^
12	Mg	229	11.25	*(2a)*	0	th.^84^
13	Al	225	10.34	*(4a)*	0	exp.^85^
14	Si	225	9.19	*(4a)*	0	exp.^86^
15	P	221	9.95	*(1a)*	0	exp.^87^
16	**S**	**166**	**9.99**	***(3b)***	**0**	**exp.**^46^
17	Cl	64	11.40	*(8 f) y = 0.31818 z = −0.37903*	0	th.^81^
18	Ar	194	12.62	*(2d)*	0	—
19	K	64	11.81	*(8d) x = −0.28496*	0	th.^88^
				*(8 f) y = 0.32494 z = 0.32775*		
20	Ca	92	12.52	*(8b) x = −0.18001 y = −0.48461 z = −0.15417*	<26	th.^89^
21	Sc	88	12.11	*(16 f) z = −0.27743 y = −0.19917 z = 0.47208*	0	exp.^90^
22	Ti	139	10.89	*(2b)*	<26	th.^91^
23	V	229	9.85	*(2a)*	0	th.^92^
24	Cr	229	9.06	*(2a)*	0	—
25	Mn	194	8.48	*(2c)*	0	exp.^93^
26	Fe	194	8.18	*(2c)*	0	exp.^94^
27	Co	225	8.15	*(4a)*	<26	exp.^95^
28	Ni	225	8.31	*(4a)*	0	th.^48^
29	Cu	225	8.67	*(4a)*	0	—
30	Zn	194	9.64	*(2c)*	0	exp.^96^
31	Ga	225	10.93	*(4a)*	0	th.^47^
32	Ge	139	11.64	*(2a)*	<26	exp.^96^
33	As	229	12.02	*(2a)*	0	th.^97^
34	Se	166	12.72	*(3b)*	0	exp.^98^
35	Br	71	13.68	*(2d)*	<26	th.^99^
36	Kr	194	15.58	*(2c)*	0	—
37	Rb	64	15.00	*(8d) x = 0.21706*	0	th.^88^
				*(8 f) y = −0.32320 z = −0.17520*		
38	Sr	62	14.96	*(4c) x = 0.17310 z = −0.07136*	0	−
39	Y	14	14.80	*(4e) x = 0.24843 y = 0.04533 z = −0.28604*	<26	exp.^100^
40	Zr	229	13.82	*(2a)*	0	exp.^101^
41	Nb	229	13.00	*(2a)*	0	th.^102^
42	Mo	229	12.48	*(2a)*	0	th.^102^
43	Tc	194	11.73	*(2d)*	0	th.^103^
44	Ru	194	11.26	*(2c)*	0	th.^104^
45	Rh	225	11.26	*(4a)*	0	—
46	Pd	225	11.61	*(4a)*	0	th.^105^
47	Ag	225	12.28	*(4a)*	0	—
48	Cd	194	13.52	*(2d)*	0	—
49	**In**	**225**	**15.07**	***(4a)***	**0**	**th.**^47^
50	Sn	139	15.89	*(2b)*	0	th.^106^
51	Sb	229	16.39	*(2a)*	0	—
52	Te	225	16.53	*(4a)*	0	th.^107^
53	I	225	17.35	*(4a)*	0	—
54	Xe	194	20.05	*(2c)*	0	—
55	Cs	194	19.00	*(2a)*	0	th.^108^
				*(2c)*		
56	Ba	194	18.73	*(2d)*	0	—
57	La	225	16.73	*(4a)*	0	exp.^109^

In the Δ*H*_EA−GS_ column, (i) *0* indicates that the EA crystal structure is lower in enthalphy than the corresponding LIT crystal structure; (ii) <*26* that the EA crystal structure is degenerate in enthalpy with the corresponding LIT crystal structure, (iii) Otherwise, it indicates the difference in enthalpy between the ground-state crystal structure and the EA-generated crystal structure, in meV/atom. In the *Ref* column, (i) *th*. (*exp*.) indicates that the literature source is computational (experimental), or (ii) *-* missing.

**Table 8:** Evolutionary algorithm database (DB_EA) at 200 GPa.

Z	Element	Space group	Volume (Å^3^/*atom*)	Wyckoff positions	ΔH_EA−GS_ (meV/atom)	Ref.
1	H	64	1.70	*(8 f) y = −0.12910 z = 0.03916*	<26	th.^76^
2	He	194	2.70	*(2c)*	0	—
3	Li	230	4.52	*(16b)*	<26	th.^51^
4	Be	194	4.33	*(2d)*	0	th.^77^
5	B	64	4.34	*(8 f) y = 0.34220 z = 0.41304*	0	exp.^78^
6	C	227	4.34	*(8b)*	0	th.^79^
7	**N**	**113**	**4.64**	***(4e) x = −0.33587 z = 0.32228***	**0**	**th.**^50^
				***(4d) z = −0.16015***		
8	O	12	5.08	*(4i) x = 0.22601 z = −0.20344*	0	th.^80^
				*(4i) x = −0.29538 z = −0.20316*		
				*(8j) x = −0.46534 y = 0.25994 z = 0.20327*		
9	F	64	5.28	*(8 f) y = −0.16484 z = −0.11903*	0	th.^81^
10	Ne	225	5.54	*(4a)*	0	exp.^82^
11	Na	62	8.00	*(4c) x = 0.32391 z = −0.08020*	<26	exp.^9^
				*(4c) x = 0.48576 z = 0.30971*		
12	Mg	229	9.02	*(2a)*	0	th.^84^
13	Al	194	8.45	*(2c)*	<26	exp.^85^
14	Si	225	7.81	*(4a)*	0	exp.^86^
15	P	191	7.90	*(1b)*	0	exp.^110^
16	S	166	8.13	*(3b)*	0	exp.^111^
17	Cl	71	9.07	*(2a)*	0	th.^81^
18	Ar	194	10.20	*(2c)*	0	—
19	K	64	9.73	*(8d) x = −0.21662*	0	th.^88^
				*(8 f) y = 0.32384 z = 0.17522*		
20	Ca	62	9.30	*(4c) x = 0.33247 z = −0.39914*	0	exp.^112^
21	**Sc**	**14**	**9.22**	***(4e) x = −0.24789 y = −0.08441 z = 0.29798***	**0**	**exp**.^90^
22	Ti	229	8.72	*(2a)*	0	th.^91^
23	V	166	8.39	*(3a)*	0	th.^92^
24	Cr	229	7.97	*(2a)*	0	—
25	Mn	194	7.57	*(2d)*	0	exp.^93^
26	Fe	194	7.29	*(2c)*	0	exp.^94^
27	Co	225	7.24	*(4a)*	0	exp.^95^
28	**Ni**	**225**	**7.34**	***(4a)***	**0**	**th**.^48^
29	Cu	225	7.57	*(4a)*	0	—
30	Zn	194	8.28	*(2c)*	0	exp.^96^
31	Ga	225	9.21	*(4a)*	0	th.^47^
32	Ge	194	9.80	*(2c)*	0	exp.^96^
33	As	229	10.15	*(2a)*	0	th.^97^
34	Se	229	10.62	*(2a)*	0	exp.^96^
35	Br	225	11.20	*(4a)*	0	th.^99^
36	Kr	194	12.63	*(2d)*	0	—
37	Rb	194	12.18	*(2a)*	0	th.^88^
				*(2d)*		
38	Sr	194	11.92	*(2d)*	0	—
39	Y	70	11.64	*(16e) x = 0.43748*	0	th.^113^
40	Zr	229	11.32	*(2a)*	0	—
41	Nb	229	11.17	*(2a)*	0	th.^102^
42	Mo	229	10.97	*(2a)*	0	th.^102^
43	Tc	194	10.35	*(2c)*	0	—
44	Ru	194	10.09	*(2c)*	0	th.^104^
45	Rh	225	10.06	*(4a)*	0	—
46	Pd	225	10.25	*(4a)*	0	—
47	Ag	225	10.69	*(4a)*	0	—
48	Cd	194	11.50	*(2c)*	0	—
49	In	139	12.68	*(2b)*	0	th.^47^
50	Sn	194	13.40	*(2c)*	0	th.^106^
51	Sb	229	13.80	*(2a)*	0	—
52	Te	225	13.96	*(4a)*	0	th.^107^
53	I	225	14.37	*(4a)*	0	—
54	Xe	194	16.24	*(2c)*	0	—
55	Cs	225	15.60	*(4a)*	0	th.^108^
56	Ba	194	15.67	*(2d)*	0	—
57	La	225	14.14	*(4a)*	0	exp.^109^

In the Δ*H*_EA-GS_ column, (i) *0* indicates that the EA crystal structure is lower in enthalphy than the corresponding LIT crystal structure; (ii) <*26* that the EA crystal structure is degenerate in enthalpy with the corresponding LIT crystal structure, (iii) Otherwise, it indicates the difference in enthalpy between the ground-state crystal structure and the EA-generated crystal structure, in meV/atom. In the *Ref* column, (i) *th*. (*exp*.) indicates that the literature source is computational (experimental), or (ii) *-* missing.

**Table 9:** Evolutionary algorithm database DB_EA at 300 GPa.

Z	Element	Space group	Volume (Å^3^/*atom*)	Wyckoff positions	ΔH_EA−GS_ (meV/atom)	Ref.
1	H	64	1.44	*(8 f) y = −0.12912 z = −0.05825*	<26	th.^76^
2	He	194	2.32	*(2d)*	0	—
3	**Li**	**73**	**3.82**	***(16 f) x = 0.12476 y = 0.12417 z = 0.37634***	**40.3**	**th.**^51^
4	Be	194	3.81	*(2c)*	0	th.^114^
5	B	64	3.93	*(8 f) y = 0.157750 z = −0.08527*	0	—
6	C	194	4.00	*(4e) z = −0.09278*	0	—
				*(4 f) z = −0.34395*		
7	**N**	**64**	**4.18**	***(8 f) y = 0.10538 z = −0.10004***	**0**	**th.**^50^
8	O	166	4.55	*(6c) z = −0.06915*	0	—
9	F	64	4.72	*(8 f) y = −0.32735 z = −0.37974*	0	th.^81^
10	Ne	225	4.91	*(4a)*	0	—
11	Na	194	6.68	*(2a)*	<26	exp.^9^
				*(2d)*		
12	Mg	229	7.84	*(2a)*	0	th.^84^
13	Al	194	7.57	*(2c)*	0	exp^115^
14	Si	225	7.00	*(4a)*	0	—
15	P	220	6.84	*(16c) x = 0.47267*	0	—
16	S	166	7.15	*(3a)*	0	th.^116^
17	Cl	139	7.90	*(2a)*	0	th.^81^
18	Ar	194	8.92	*(2c)*	0	—
19	K	194	8.47	*(2a)*	0	th.^88^
				*(2d)*		
20	Ca	62	8.07	*(4c)*	0	—
21	Sc	70	7.81	*(16 g) z = 0.31244*	<26	exp.^90^
22	Ti	229	7.61	*(2a)*	0	exp.^117^
23	V	229	7.57	*(2a)*	0	th.^92^
24	Cr	229	7.28	*(2a)*	0	—
25	Mn	194	6.91	*(2d)*	0	—
26	Fe	194	6.73	*(2c)*	0	exp.^94^
27	Co	225	6.67	*(4a)*	0	—
28	Ni	225	6.73	*(4a)*	0	—
29	Cu	225	6.91	*(4a)*	0	—
30	Zn	194	7.49	*(2d)*	0	—
31	Ga	225	8.27	*(4a)*	0	—
32	Ge	194	8.74	*(2c)*	0	exp.^96^
33	As	229	9.07	*(2a)*	0	—
34	Se	139	9.44	*(2a)*	0	exp.^96^
35	Br	225	9.91	*(4a)*	0	th.^118^
36	Kr	194	11.09	*(2d)*	0	—
37	Rb	225	10.79	*(4a)*	<26	th.^88^
38	Sr	194	10.61	*(2c)*	0	—
39	**Y**	**71**	**10.24**	***(2c)***	**110.1**	**th.**^52^
40	Zr	229	10.05	*(2a)*	0	—
41	Nb	229	10.04	*(2a)*	0	th.^102^
42	Mo	229	9.99	*(2a)*	0	th.^102^
43	Tc	194	9.52	*(2c)*	0	th.^103^
44	Ru	194	9.32	*(2d)*	0	th.^104^
45	Rh	225	9.30	*(4a)*	0	—
46	Pd	225	9.43	*(4a)*	0	—
47	Ag	225	9.76	*(4a)*	0	—
48	Cd	194	10.52	*(2c)*	0	—
49	In	139	11.38	*(2a)*	0	th.^47^
50	Sn	194	12.01	*(2d)*	0	—
51	Sb	229	12.37	*(2a)*	0	—
52	Te	225	12.49	*(4a)*	0	—
53	I	139	12.73	*(2b)*	0	—
54	Xe	194	14.19	*(2c)*	0	—
55	Cs	225	13.87	*(4a)*	0	—
56	Ba	194	14.03	*(2c)*	0	—
57	La	139	12.61	*(2a)*	0	—

In the Δ*H*_EA−GS_ column, (i) *0* indicates that the EA crystal structure is lower in enthalphy than the corresponding LIT crystal structure; (ii) <*26* that the EA crystal structure is degenerate in enthalpy with the corresponding LIT crystal structure, (iii) Otherwise, it indicates the difference in enthalpy between the ground-state crystal structure and the EA-generated crystal structure, in meV/atom. In the *Ref* column, (i) *th*. (*exp*.) indicates that the literature source is computational (experimental), or (ii) *-* missing.

**Table 10:** Database of structures (DB_MISS) at different pressures.

Pressure (GPa)	Z	Element	Space group	Volume (Å^3^/*atom*)	Wyckoff positions	ΔH_LIT−GS_ (meV/atom)	Ref.
100	16	S	bco	*N/A*	*N/A*	*N/A*	exp.^46^
100	49	In	bct	*N/A*	*N/A*	*N/A*	th.^47^
200	7	N	32	*N/A*	*N/A*	*N/A*	th.^50^
200	21	Sc	*N/A*	*N/A*	*N/A*	*N/A*	exp.^90^
200	28	Ni	229	7.34	*(2a)*	173.2	th.^48^
300	7	N	32	*N/A*	N/A	*N/A*	th.^50^

The Δ*H*_EA-GS_ column represents the difference in enthalpy between the ground-state crystal structure and the LIT crystal structure, in meV/atom. In the *Ref* column, (i) *th*. (*exp*.) specifies whether the literature source is computational (experimental). N/A indicates that the available information is too incomplete to completely characterize the structure (*unreproducible*).

## Data Records

Our *HEX* database^31^ comprises the three sub-databases described below. Details of the relative structures are reported in the Tables 2–10; the corresponding CIF files can be found at figshare 10.6084/m9.figshare.c.7119778.v1.

- **DB_GS (Database Ground-State)**: The main sub-database includes the ground-state structure for each element at 0, 100, 200, 300 GPa, obtained by comparing the result of our evolutionary crystal structure prediction runs (EA structures) with the structures obtained from the screening of the literature (LIT structures), when available.
  The columns of Tables 2–5 contain the atomic number *Z*, element symbol, space group, unit cell volume (per atom), and the Wyckoff positions of the ground-state structures; the column *Source* specifies whether the lowest-energy structure was found through EA runs (ea), or in literature (lit); an asterisk (*) indicates that the EA-generated structure agrees with the literature, while a dash (−) indicates that we could not find a literature reference for the relevant element and pressure (*unreported* structures). In cases where the difference in enthalpy between the EA-generated structure and the literature one was below 26 meV/atom, the structures were considered to be *degenerate*. In the following, structures for which literature and EA results are the same are named *matching*, while those different are named *mismatching*. The column *Ref* reports the literature reference.
- **DB_EA (Database Evolutionary Algorithm)**: This database contains the results of our evolutionary algorithm searches for every combination of element and pressure considered. The main results are summarized in Tables 6–9. The columns contain the atomic number *Z*, element symbol, unit cell volume (per atom), the Wyckoff positions, the relative enthalpy compared to that of the ground-state structure. The relative enthalpy ΔH_EA-GS_ is zero in cases where the EA predicts the lowest-enthalpy structure, and < 26 when the difference between EA and LIT ground-state structure is smaller than 26 meV/atom, and positive otherwise. The column *Ref* reports the literature reference. We indicate in bold-face the entries for which the EA predictions are *unsuccessful*, i.e. cases in which the EA-predicted structures are neither *matching* nor *degenerate* with avaiable experimental data.
- **DB_MISS (Database Miss)**: This database contains the list of literature structures less stable than EA-generated ones, and hence not included in the ground-state tables. The structures for all pressures are grouped into a single table – Table 10. The columns contain the atomic number *Z*, the element symbol, the space group when available, the unit cell volume (per atom), the Wyckoff positions, the enthalpy relative to the ground-state, and the literature reference, together with the indication whether the literature reference is theoretical or experimental. We also indicate explicitely when literature references did not report enough structural information to allow for a comparison with EA-generated structures (*non-reproducible* in the following).

**Fig. 1:**
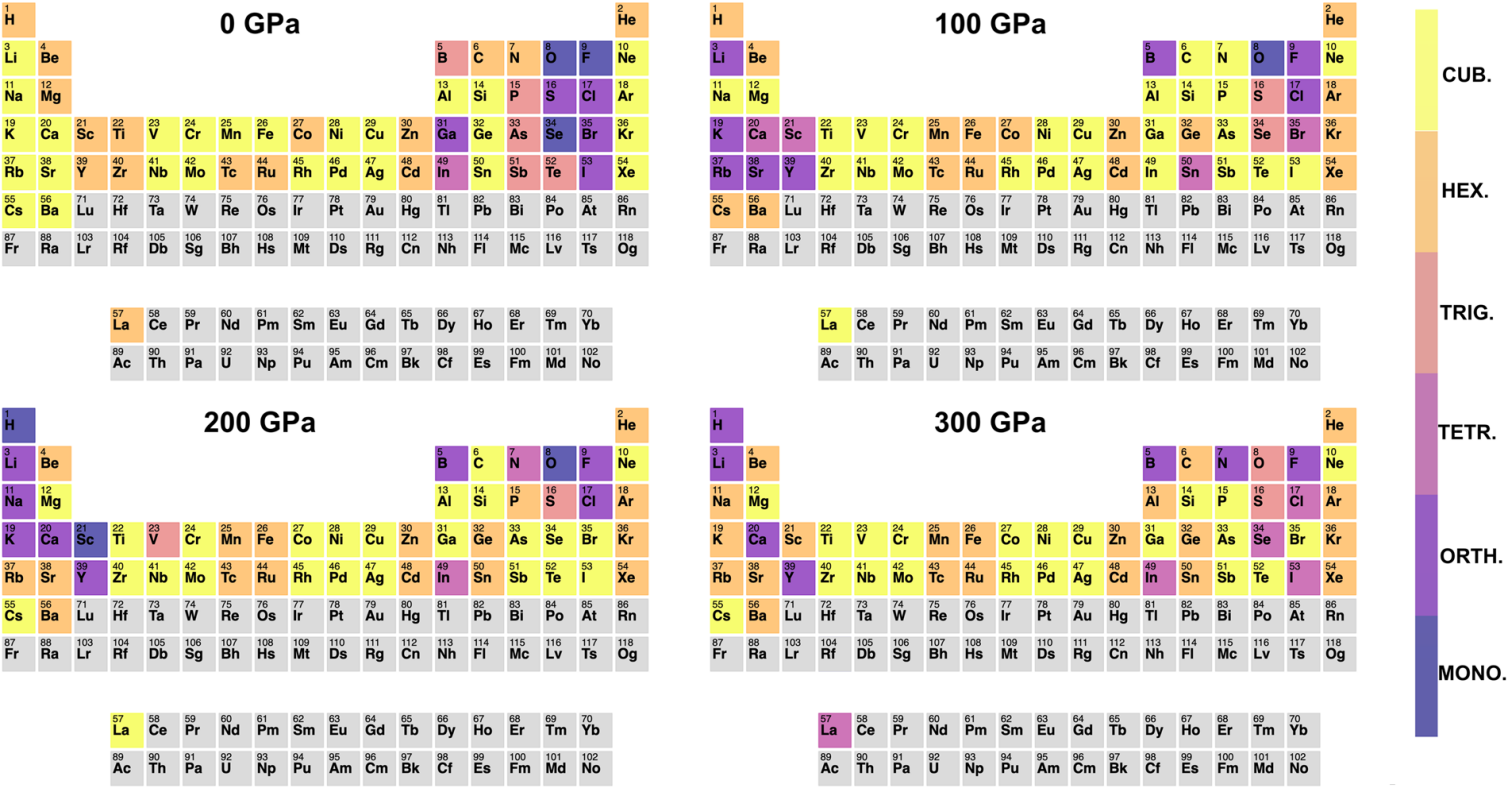
Lattice systems for the ground-state structures at 0, 100, 200 and 300 GPa. The colorbar indicates the Bravais lattice: (i) *MONO*.: space group 3–15 (monoclinic); (ii) *ORTH*. space group 16–74 (orthorombic); (iii) *TETR*. space group 75–142 (tetragonal); (iv) *TRIG*. space group 143–167 (trigonal); (v) *HEX*. space group 168–194 (hexagonal); (vi) *CUB*. space group 195–230 (cubic). The plots have been prepared with the *ptable_trends* program^119^.

In Fig. 1 the trends in the evolution of the crystal structure of the elements with pressure are summarized in graphical form. The four periodic tables indicate, for each element, the lattice system of the ground-state crystal structure at pressures of 0, 100, 200 and 300 GPa: Monoclininc (3–15), Orthorombic(16–74), Tetragonal (75–142), Trigonal (143–167), Hexagonal (168–194), and cubic (195–230). Bravais lattice types are indicated by a color scale, from purple to yellow.

The figure shows that for most elements the evolution of the crystal structure with pressure does not follow the naïve expectation that all matter should become more homogeneous under pressure by adopting more close-packed structures. In fact, except for transition metals and noble gases, which adopt either face- or body-centered cubic or hexagonal close-packed structures over the whole range of pressures, other elements undergo a series of transitions, sometimes leading to very complex structures, which may exhibit lower symmetries than ambient-pressure ones. The observed deviation from hard-sphere close-packing at high-pressure can originate from different physical mechanisms: charge localization in interstitial sites (electride behavior), in alkali and alkali metals; stabilization of polymeric or molecular phases, in pnictides, chalcogenides and halides; repopulation of atomic orbitals, leading to change in formal valence, as in III and IV-row elements^16,18^.

## Technical Validation

Validation is an intrinsic part of our work, which comprised a thorough comparison of the results of extensive evolutionary algorithm searches, sampling over 70.000 structures, with available literature data.

**Fig. 2:**
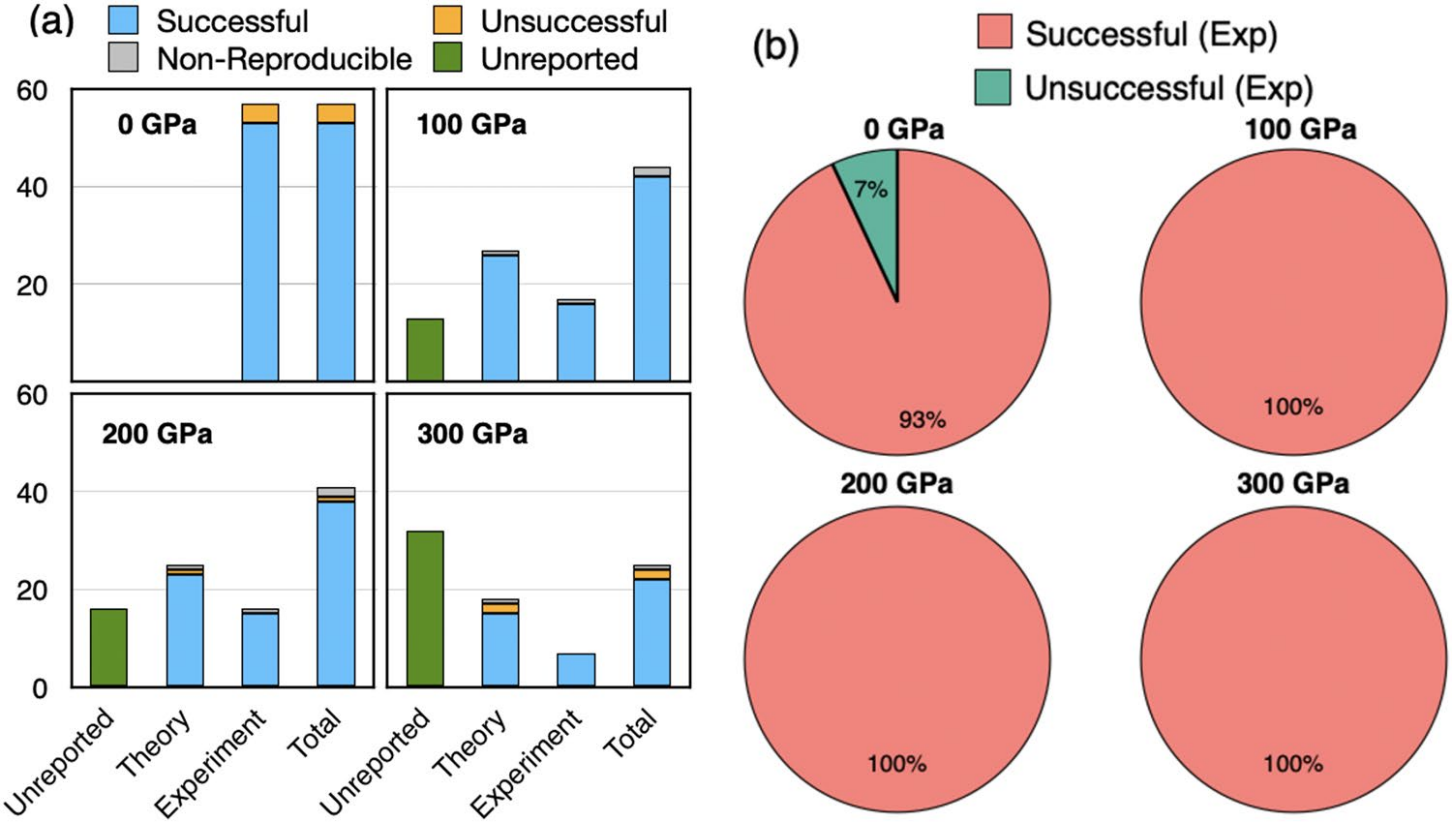
(**a**) The bar chart gives a breakdown of the whole dataset for different pressure into structures (i) Unreported (green) and (ii) reported in literature, divided into *Experiment* and *Theory* category. The color of the bar indicates whether EA-predicted structures exactly match or are degenerate with available literature data – *successful* (blue)/*unsuccessful* (yellow). (**b**) Pie chart displaying the fraction of *successful* EA predictions, considering only fully characterized experimental structures. Red (green) represents the *successful* (*unsuccessful*) cases for all pressures.

Figure 2 summarizes the current status of knowledge of high-pressure (HP) structures and presents a comparison with EA-generated structures. The bar chart indicates for each pressure the amount of information available in literature on the structures of the first 57 elements. Structures for each element are divided into *Unreported*, *Theory*, *Experiment*, depending on whether any information is available in literature, and if the source is an experiment or a theoretical prediction – The column *Total* is the sum of *Theory* and *Experiment*. The bars are colored to indicate whether our EA-prediction runs were *succesful/unsuccesful* in reproducing literature data. A *succesful* prediction implies that the EA-predicted structure is either exactly *matching* the literature structure or *degenerate* with it to within 300 K (26 meV). Cases in which literature information did not contain enough data to fully reproduce the structures are indicated as *Non-reproducible*.

While at ambient pressure the structures of all these elements have been experimentally determined and are collected in American Mineralogist Crystal Structure Database^45^, as pressure increases fewer and fewer experimental reports of high-pressure elemental phases can be found. For example, at 300 GPa, experimental information is available for only about 15% of the 57 elements considered in this work; about twice the same amount of structures can be recovered from theoretical predictions, but for more than 50% the structure is *unreported*.

In general there is a remarkable agreement between our EA predictions and experiment. Moreover, we find that for most cases where we could not identify any literature reference, our EA calculations predict that the elements will retain the same crystal structure measured at lower pressures. In the rare cases in which we observe a disagreement between EA predictions and experiments *a posteriori* it is easy to find very plausible explanations, discussed in the following.

In the right panel of Fig. 2 we use a pie chart to quantify the success rate of EA predictions. The comparison in this case involves only cases for which full experimental information is available. On average, we find that ~ 98% of the EA predictions were *succesful*, i.e. EA either predicted the same structure as experiment (*matching* structures), or a structure *degenerate* with it to within 26 meV.

- **Ambient pressure (0 GPa)**: Of the 57 papers found in literature for 0 GPa, 36 reports are *matching* with our studies. Of the remaining 21 *mismatching* cases, 17 are *degenerate* in enthalpy. This means that 53 structures can labeled as *succesful*.
  In a few cases, the original mismatch between EA predictions and experiment was eliminated including corrections to the standard GGA functional used for all our calculations. In particular, for Br and I, marked with daggers in the tables, the experimental ground-state structures become degenerate in enthalpy with our calculated structures after adding Van-Der-Waals corrections. While experimentally these elements form molecular crystals, the structures we predict contain zig-zag polymeric chains. Since the two types of structures are almost degenerate in energy, it is conceivable that, depending on the activation energy and temperature dependence of the polymerization, also polymeric structures might be experimentally realizable.
  In order for the EA predictions to match experiments for for Fe, Co and Ni, we had to include spin polarization in the calculations. These enetries are marked with asterisks in the table.
  Of the four *unsuccessful* structures, B, S and Mn have a ground-state characterized by cells much larger than the 8 atoms cell we considered for our EA searches, while for tellurium, we believe that the source of the discrepancy may be a substantial role of spin-orbit effects, which are neglected in our calculations.
  In synthesis, at 0 GPa group 93% of the EA predictions can be defined *successful*, according to our criteria.
- **100 GPa Group**: Of the 44 structures reported in literature for 100 GPa, our EA predictions are *matching* for 34 elements. Of the remaining 10 *mismatching* cases, 8 are *degenerate* in enthalpy. For the remaining two elements, S and In, literature references did not contain enough information to fully reconstruct the structures, only the Bravais lattices – bco for S^46^ and bct for In^47^. Hence, they should be classified as *unreported*.
  At 100 GPa, our EA structures are *successful* in reproducing the literature data in 100% of the cases where complete experimental information was available.
- **200 GPa Group**: Of the 41 papers found in literature for 200 GPa, our EA predictions are *matching* in 34 cases. Of the remaining 7 *mismatching* cases, 4 are *degenerate*. We have not been able to gather enough information to perform calculations on the reported phases for N and Sc, which should then be classified as *unreported*. The EA-predicted structure for Ni (fcc) is more stable than the bcc phase predicted by Belashenko *et al*.^48^. Including spin-polarization in the calculation does not modify this result. It is likely that strong correlation effects may solve the discrepancy^49^.
  At 200 GPa, taking into consideration only fully experimentally-determined structures, *successful* predictions are hence 100% of the total.
- **300 GPa Group**: Our EA predictions *match* 18 of the 25 structures reported in literature for 300 GPa. Of the 7 *mismatching* cases, 4 are *degenerate* in enthalpy. Of the remaining elements, the reference reported for N did not contain enough information to fully determine the crystal structure^50^, and should then be considered *unreported*. For Li^51^ and Y^52^, the structures we obtained were found to be less stable than theoretical predictions in literature, which however employed much larger unit cells.

In summary, at 300 GPa, our EA structures reproduced literature results in 92% of the cases. Taking into consideration only fully-determined experimental structures, the fraction of *successful* predictions rises to 100%.

An exciting outcome of our work is that evolutionary crystal structure predictions based on Density Functional Theory are extremely accurate: on average 96% of structures available in literature were predicted correctly (98% considering only fully-determined experimental structures). In all but two cases where EA-predicted structures could not reproduce the ground-state structures from the literature, we could attribute this either to physical effects not included in our original computational setup (vdW interactions, magnetism, spin-orbit coupling) or to the choice of a too small unit cell. The only two cases for which we could not find a simple explanation are Te at ambient pressure, and Ni at 200 GPa.

**Fig. 3:**
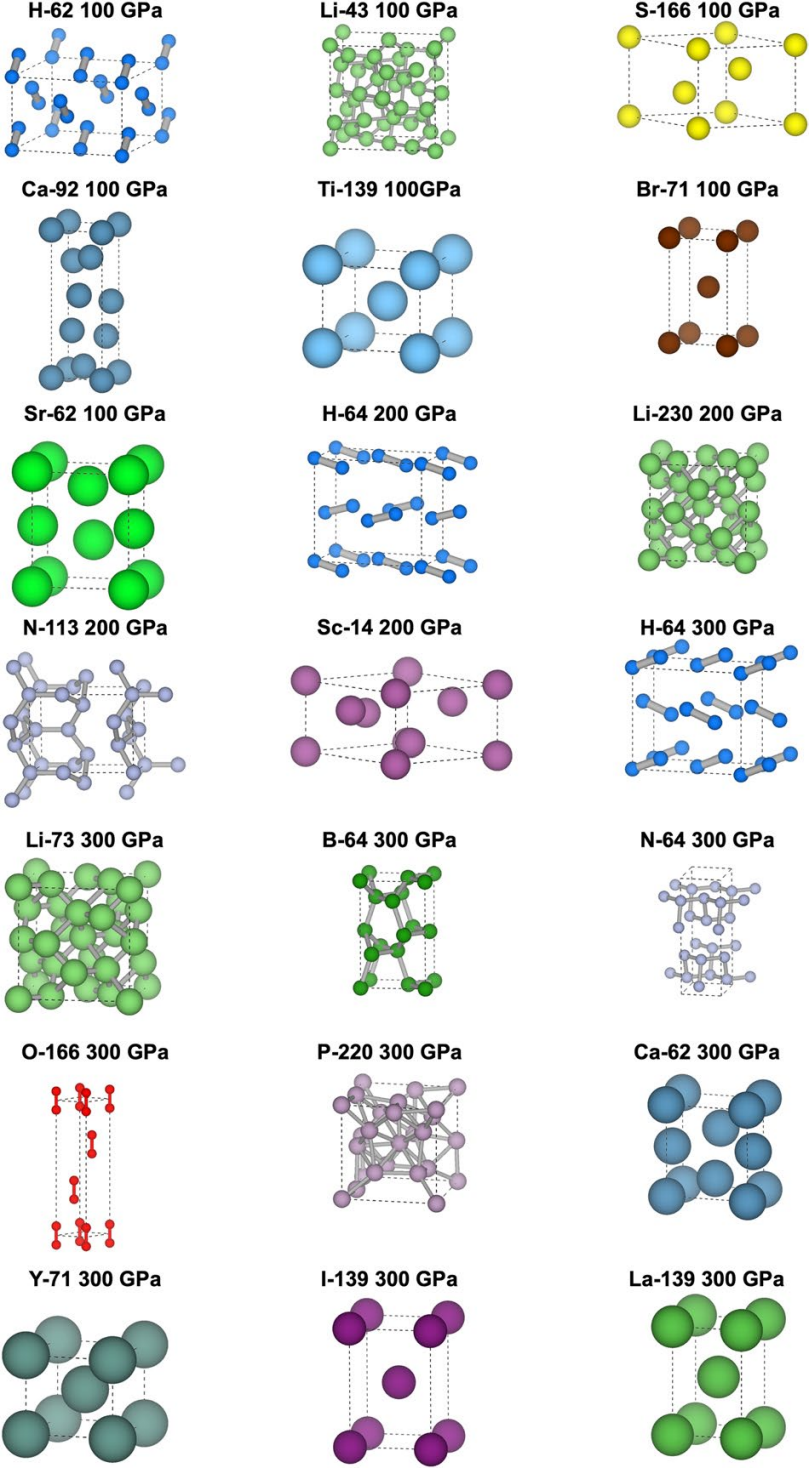
EA-predicted crystal structures for elements and pressure where the experimental information is either completely missing, or too incomplete to reconstruct the structure. We leave out trivial cases in which the structure is a monoatomic *fcc*, *bcc* or *hcp* one. Structures are labelled as: Element-Space Group number and pressure.

In Fig. 3, we show EA-generated crystal structures for the 21 cases which we believe may be of interest for future studies, labeled with their element, space group number, and pressure. In all these cases, experimental information is either not available at all, or too incomplete to completely determine the structure. We decided to not show, however, trivial cases in which EA predicted is a monoatomic *bcc*, *fcc* or *hcp* ground-state structures.

Of the structures shown in figure, hydrogen and oxygen tend to form such strong bonds, that they form molecular crystals up to the the highest pressure considered in this work. Nitrogen and boron, whose covalent bonds are more prone to frustration, form complex crystalline polymers. Lithium and phosphorous form complex, high-symmetry phases with large unit cells. Heavier elements tend to form less exotic structures, mainly tetragonal distorsions of cubic structures. We note that the qualitative behavior is consistent with what is observed in other elements, where high-pressure data is available.

## Usage Notes

Data are stored on figshare 10.6084/m9.figshare.c.7119778.v1, in two separate compressed zip archives. The first archive - *HEX.zip* - contains three folders, one for each of the databases described in the text (GS, EA, MISS). Moreover, each folder contains four sub-folders, one for each pressure. The sub-folders contain files in the standard Crystallographic Information File (cif), named as *ELEMENT_PRESSURE_DATABASE.cif* (*DATABASE* = *GS, EA, MISS*). The second archive – *Evolutionary.zip* – contain the input files used for the evolutionary prediction runs (USPEX input files + example of VASP INCAR files).

## Data Availability

All calculations described in the paper have been carried out using the *Vienna ab-initio Simulation Package* (VASP), v 6.3.0, for DFT total energies, forces, and structural relaxations, the *Universal Crystal Structure Predictor* (USPEX), v 10.5, for crystal structure searches. No custom code was used during this study for the curation of the *HEX* database^31^. The methods section contains all details needed to reproduce the calculations.
